# Prediction of the Nitrogen Content of Rice Leaf Using Multi-Spectral Images Based on Hybrid Radial Basis Function Neural Network and Partial Least-Squares Regression

**DOI:** 10.3390/s22228626

**Published:** 2022-11-09

**Authors:** Yawen Wu, Saba J. Al-Jumaili, Dhiya Al-Jumeily, Haiyi Bian

**Affiliations:** 1 Faculty of Electronic Information Engineering, Huaiyin Institute of Technology, Huai’an 223003, China; 2 Laboratory of Climate-Smart Food Crop Production, Institute of Tropical Agriculture and Food Security, Universiti Putra Malaysia (UPM), Serdang 43400, Selangor, Malaysia; 3 School of Computer Science and Mathematics, Faculty of Engineering and Technology, Liverpool John Moores University, Liverpool L3 5UX, UK

**Keywords:** image processing, predication, artificial neural network, rice leaves

## Abstract

This paper’s novel focus is predicting the leaf nitrogen content of rice during growing and maturing. A multispectral image processing-based prediction model of the Radial Basis Function Neural Network (RBFNN) model was proposed. Moreover, this paper depicted three primary points as the following: First, collect images of rice leaves (RL) from a controlled condition experimental laboratory and new shoot leaves in different stages in the visible light spectrum, and apply digital image processing technology to extract the color characteristics of RL and the morphological characteristics of the new shoot leaves. Secondly, the RBFNN model, the General Regression Model (GRL), and the General Regression Method (GRM) model were constructed based on the extracted image feature parameters and the nitrogen content of rice leaves. Third, the RBFNN is optimized by and Partial Least-Squares Regression (RBFNN-PLSR) model. Finally, the validation results show that the nitrogen content prediction models at growing and mature stages that the mean absolute error (*MAE*), the Mean Absolute Percentage Error (*MAPE*), and the Root Mean Square Error (*RMSE*) of the RFBNN model during the rice-growing stage and the mature stage are 0.6418 (%), 0.5399 (%), 0.0652 (%), and 0.3540 (%), 0.1566 (%), 0.0214 (%) respectively, the predicted value of the model fits well with the actual value. Finally, the model may be used to give the best foundation for achieving exact fertilization control by continuously monitoring the nitrogen nutrition status of rice. In addition, at the growing stage, the RBFNN model shows better results compared to both GRL and GRM, in which *MAE* is reduced by 0.2233% and 0.2785%, respectively.

## 1. Introduction

With the increasing proportion of the rice industry in Malaysia industries and its important position in world food security, improving the output and quality of rice has become an important issue [1,2,3,4,5,6]. Nitrogen (N) is an essential element for the growth and development of rice, which directly affects the rice leaves and has a decisive effect on the yield and quality of rice [7,8,9,10,11]. Most cultivated crops have accelerated leaf senescence, decreased chlorophyll content, and decreased crop yields due to nitrogen deficiency [12,13].

Furthermore, the N deficiency significantly hinders crop growth. Thus, noticing and solving the problem on time helps in preventing crop losses; it is also helpful to know what causes N deficiency in plants and avoid that correspondingly, and early problem detection facilitates a successful outcome. Plants with N deficiency are thin, pale, subject to chlorosis, and produce poor fruits. By knowing how to control N deficiency in plants with organic and chemical methods, farmers can save yields. Remote sensing assists agriculturalists in identifying the problem early [14,15].

Moreover, it is important for chlorophyll production, which is essential if plants are photosynthesized to acquire their nutrition. Furthermore, it is also a component of amino acids, DNA, membrane proteins, enzymes, the majority of coenzymes, auxins, and cytokinins, making it essential for the growth of cells in plants; this is why nitrogen deficiencies, reduced growth, and reduced yields are crucial to prevent and control. Instead, the optimal nitrogen fastening and N supply enable healthy development and full capability for crop production. A significant issue with N insufficiency is the low protein content of grains such as rice, wheat, and corn [16]. Furthermore, pertinent research has demonstrated that the optimal fertilization amount of rice may be estimated using the nutritional detection of the nitrogen content of rice and that accurate fertilization of rice can subsequently be achieved [17,18,19,20]. However, utilizing conventional chemical analysis and diagnosis techniques has the drawbacks of being time-consuming, labor-intensive, damaging to plants, and slow diagnosis, and it is difficult to implement fertilization recommendations quickly and effectively. The implementation of new technologies and methods for crop nutrition diagnostics has increasingly come into the focus of research in recent years due to the rapid growth of information technology [21,22]. To optimize the administration of nitrogen fertilizer, numerous researchers have recently developed various detecting algorithms for the nutritional state of plants. A new technology for measuring plant physiological condition was proposed by several researchers [11,23], employing leaf and leaf scale sun-induced chlorophyll fluorescence to calculate the nitrogen content and photosynthetic nitrogen usage efficiency of wheat leaves. It is essential to efficiently acquire data on the nutrients found in leaf nitrogen in order to provide accurate fertilization advice and improve the development of modern agriculture. Chemical technologies are used in traditional nutrient information detection, but they are labor-intensive, expensive, and easily pollute the environment, making them unsuitable for the development needs of contemporary precision agriculture. Because variations in the amount of nitrogen fertilizer may alter the physiology and morphology of crop leaves, which in turn may alter the properties of the crop’s spectrum reflectance [24]. Furthermore, much progress has been made in researching the characteristics of spectral changes and nitrogen content at home and abroad. The main results include the research on the sensitive bands of plant nitrogen content, mainly in the near-infrared waveband [25,26], red light waveband, and green light waveband [27], Visible light band [28].

In order to predict the leaf nitrogen concentration of maize plants, a number of authors [12,13,29,30,31,32] suggested a method using machine learning technology and multispectral imaging. This study offers a theoretical scientific foundation for identifying and diagnosing plant growth status. Another researcher [12,33] used the drone’s camera to capture multispectral photos and developed a new technique to calculate the nitrogen content of RL by running spectral analysis algorithms on the photographs. The sensitive level of nitrogen was created based on the original spectral reflectance of rice leaves, which is more sensitive to changes in nitrogen. Digital image processing technology and machine vision in the visible light spectrum have been widely used in crop production, but they suffer from complicated operations, high costs, small scope of application, and unfavorable options [13].

In order to explore a low-cost and highly popular method for detecting leaf nitrogen content in rice, this paper applies digital image processing technology to construct prediction models of rice leaf color characteristic parameters, leaf area, and leaf nitrogen content in different stages under natural light [22], and the multi-scale retinex (MSR) algorithm eliminates the impact of illumination [26]. The rapid detection of nitrogen content in rice leaves can provide a theoretical basis for precise fertilizer management in orchards [24].

This paper uses novelty to improve the grid technique to measure the area of rice leaves. The neural network consists of the input layer, which is generally historical load data for predicting nitrogen content; the hidden layer is between the input and output layers and cannot be observed outside the system. The output unit realizes the output of the system processing results to predict the required data. Furthermore, the rice leaf field is measured by the number of pixels. If the number of pixels of the measured leaf and the actual area of the pixel is known, the leaf area can be obtained using the proportional relationship. Finally, the proposed model was validated in comparison to GRL and GRM, which were used to build the model.

## 2. Materials and Methods

### 2.1. Data Collection

**Figure 1:**
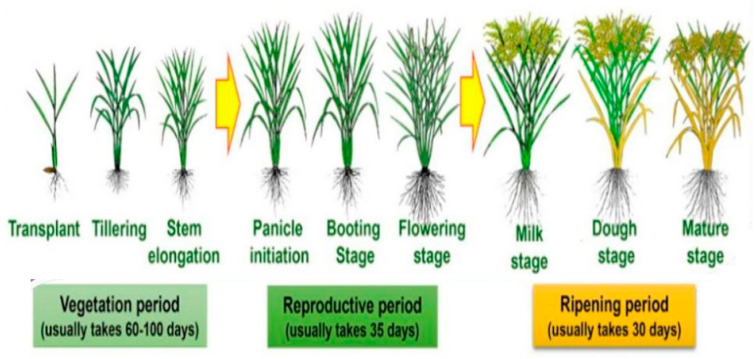
Rice plant growth stages.

Figure 1 shows the rice’s vegetative and reproductive growth stages. This paper focuses on Basmati-370 rice which has a special aroma and flavor, along with the fine quality long grain. In addition, a fertilizer called urea (which contains 46% nitrogen) should be used on crops as needed. When used and maintained correctly, it is a fertilizer for flooded soils that is reasonably inexpensive, has a high nitrogen analysis, and is effective. It can be administered to rice 3–4 weeks after transplanting and again 7–8 weeks later.

**Figure 2:**
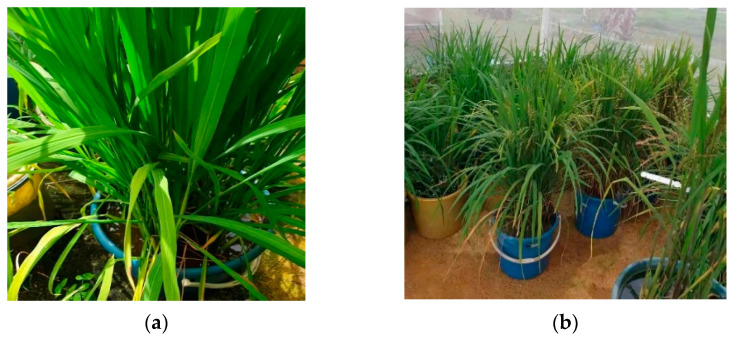
The original image of the rice leaves. (**a**) Stem elongation stage, (**b**) Flowering stage.

The experiment was carried out at University Puta Malaysia, UPM, which is situated between 3°02′ North latitude and 101°42′ East longitude and an altitude is 31 m above sea level (Figure 2). The high temperature, humidity, and sufficient rainfall characterize its tropical climate. Thus, the tropical climate challenged the plant’s implementation, which featured high humidity and significant rains, adequate sunshine, and low winds. The rice was planted from July to November 2020 in the test University Putra Malaysia, and random selection was used to choose the test sample from fields with moderate growth, a comparable quantity of buckets, and no disease or insect pests. In the experiment, 90 samples of two-stage (Stem elongation stage and Flowering stage) rice were gathered. For each type of leaf, 45 samples in the center of the outer perimeter that was flat, had no surface damage and had healthy physiological conditions were selected as samples. After the sample collection is completed, 10 leaves are randomly selected each time and placed on the calibration board, as shown in Figure 2.

### 2.2. Image Processing

RBFNN model used rice leaf area, leaf color parameters, and leaf nitrogen content in different stages to predict the nitrogen content. The number of pixels on and inside the boundary of the target area of a single paper image, as well as the pixels extracted from the segmented leaf image.

For the measurement of the pixel area value, the total number of pixels occupied by the complete segmented image is taken, and the actual area of the complete segmented image can be obtained as

(1) $$A=\sum_{i=1}^{m}\sum_{j=1}^{n}f(x,y)$$

where *m* and *n* are the sizes of the segmented complete target area.

The mean value of all the pixels in the G channel of the rice leaf image in stages of growing and maturing is extracted by

(2) $$A=\frac{x_{1}+x_{2}+\ldots \;x_{n}f_{n}}{f_{1}+f_{2}+\ldots +f_{n}}$$

where *A* is the mean value of pixels in the G channel; *x_n_* is the value of the G channel; *f_n_* is the number of pixels in the interval.

The *K* actual value and the pixel value scaling ratio, as defined in Equation (3),

(3) $$K=\frac{A}{L}$$

where *L* is the pixel value of the calibration board in the pixel coordinate system.

The actual value of the feature size of the target as,

(4) F=K∗M

where *M* is the known size of the calibration board and *K* is the feature size of the target item to be measured in the same image.

The calibration coefficient pixel area can be obtained as,

(5) $$K_{C}=\frac{S_{1}}{S_{2}}$$

where *S*_1_ is the area of a square in the calibration RL; *S*_2_ is the number of pixels occupied by a single square in the calibration board in the image, that is, the pixel area.

Whereas the performance evaluation on the RBFNN model was constructed to predict the nitrogen content as

(6) $$Performance\;Parameters=\left\{\begin{array}{c} MAE=\frac{1}{S}\overset{S}{\underset{i=1}{\sum }}\left|y_{i}-\bar{y_{l}}\right|\\ RMSE=\sqrt{\frac{1}{S}\overset{S}{\underset{i=1}{\sum }}{\left(y_{i}-\bar{y_{l}}\right)}^{2}}\\ MAPE=\frac{1}{S}\overset{S}{\underset{i=1}{\sum }}\frac{\left|y_{i}-\bar{y_{l}}\right|}{\bar{y_{l}}} \end{array}\right.$$

where S is the sample size, $y_{i}$ is the actual value of leaf nitrogen content, and $\bar{y_{i}}$ is the models’ predicted value; and three performance indicators of the mean absolute error (*MAE*), the mean absolute percentage error (*MAPE*), and the root mean square error (*RMSE*). In order to further verify the estimation accuracy of the artificial neural network model by validating used the model and using error rates as,

(7) $$E=\frac{sn_{2}-sn_{1}}{sn_{1}}\times 100\%$$

where E denotes the error rate; $sn_{1}$ is the actual measured value of the nitrogen content of rice leaves, g/kg; $sn_{2}$ predicts the value of the nitrogen content of rice leaves, g/kg. The sample data are often not a simple linear relationship in actual data analysis, and there may be a nonlinear relationship.

### 2.3. Method

In order to make linearly inseparable data linearly separable, this study uses radial basis kernel function (RBF) combined with Partial Least-Squares Regression (PLSR) [34], to build a nonlinear model to improve the accuracy of the prediction model. The nonlinear kernel function used is the Gaussian function commonly used in the radial basis function [26], representing a real-valued function whose value depends only on the distance from the origin. It is a commonly used nonlinear kernel function in regression algorithms, as seen in Equation (8),

(8) $$k\left(x,x^{\prime }\right)=\exp\left(-\frac{{\left\|x-x^{\prime }\right\|}_{2}^{2}}{2\sigma^{2}}\right)$$

where: ${\left\|x-x^{\prime }\right\|}_{2}^{2}$ is the squared Euclidean distance between the feature vectors; *σ* is a free parameter. Continuously optimizing the threshold and weighting value can effectively improve the accuracy and validity of the RBF neural network. When optimizing the RBF neural network using the PLSR algorithm, first determine the number of iterations, population size, crossover probability, and mutation probability, and perform population initialization. The weighted value and threshold output obtained by initialization is compared with the training value to determine the individual fitness [35] as in Equation (9),

(9) $$F=k\left[\sum_{i=1}^{n}abs\left(y_{i}-o_{i}\right)\right]$$

where *F* is absolute error; *i* is the current node number, *n* is the number of output nodes, *y_i_* is the original output data of the *i*-th section, and $o_{i}$ is the network prediction data of the *i*-th section.

This research mainly selects the roulette method that uses the fitness ratio as the selection method; that is, the smaller the fitness value, the higher the selection probability. Finally, the network structure is established according to the population initialization, and crossover operations and mutation operations are performed. Therefore, the main process is to design an RBFNN-PLSR model as the prediction model of leaf nitrogen content as follows:

First, the rice leaf image should have the noise removed using adaptive filtering, after which the leaf area should be retrieved from the image using segmentation, and finally, the extracted leaves should be processed morphologically.

Second, the edge of the graph paper is extracted based on an improved canny algorithm, the closed operation fills the discontinuity, and then the hole is removed by the area filling. The grid image of the calibration plate is segmented based on the RGB color feature information.

**Figure 3:**
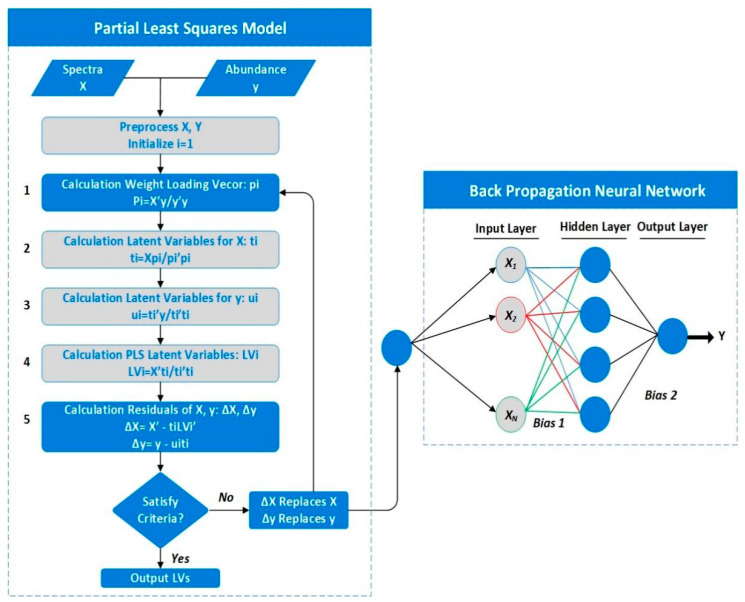
The proposed RBFNN is optimized by and Partial Least−Squares Regression model.

Finally, the connected component is extracted and marked to the predicted value of the RBFNN model predicted leaf nitrogen content (g/kg), compared to the genotype-restricted likelihood method (GRL) and General regression method (GRM). Figure 3, RBFNN-PLS model demonstrates how latent variables are determined by processing spectra.

## 3. Results and Discussion

The Kinect RGB-D camera from Microsoft^®^ Company (Redmond, WA, USA) is used to take color and depth pictures of plants. In the building’s monitoring system for plants to take pictures, a laptop computer is attached to a tripod-mounted Kinect camera. A 12 V Li-On battery powers the Kinect camera. The Kinect camera could capture images with a resolution of 640 × 480 pixels for depth and 1280 × 960 pixels for color. The resolutions of the RGB and depth images from Kinect are set to 640 × 480 pixels in order to align the color image with the depth image. To capture the optimal image, the Kinect camera and tripod are positioned 100 cm away from the plants in a horizontal direction, at a height of 130 cm, and at a 30° downward angle.

Once the model and the scenario have been described, the main results and analysis are presented in this section. Table 1 shows the selected rice varieties studied below. A total of 90 samples were collected at two different stages from each rice variety, 45 samples for the growing and mature stages. The collected sample was divided into two groups, as 35 samples were used for the training process and 10 samples for testing. Figure 2 shows the training sample of mean and variance of the overall color of the rice leaf changes dynamically in different stages.

**Figure 4:**
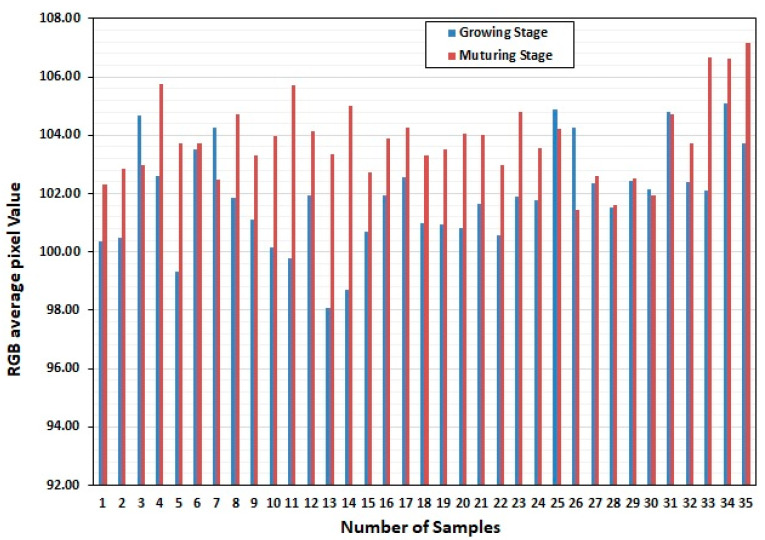
Distribution interval of pixel points in different stages in the G channel.

Figure 4 shows that the mean change of the two stages is consistent with the changing trend of leaf nitrogen content; the variance of the rice-growing stage is slightly smaller than that of the mature stage. The variance at the mature stage is slightly smaller than in the rice-growing stage. The leaf color of the mature stage is the most uneven, and the leaf color of the mature stage is the best.

**Figure 5:**
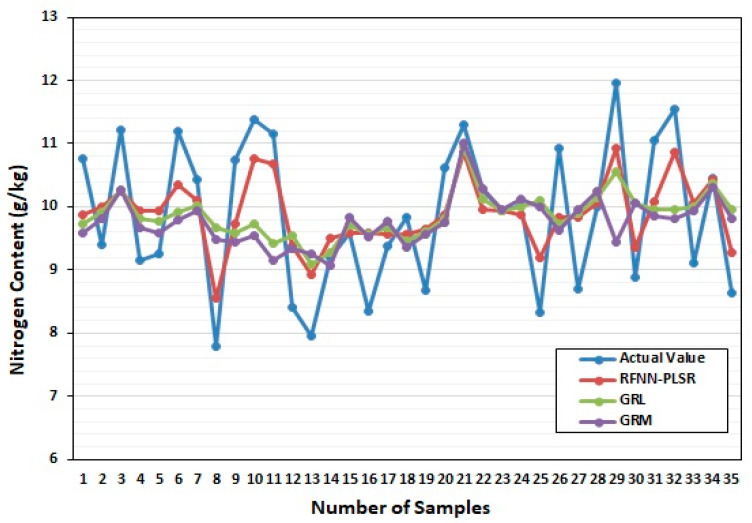
Training model during rice growing stage.

**Figure 6:**
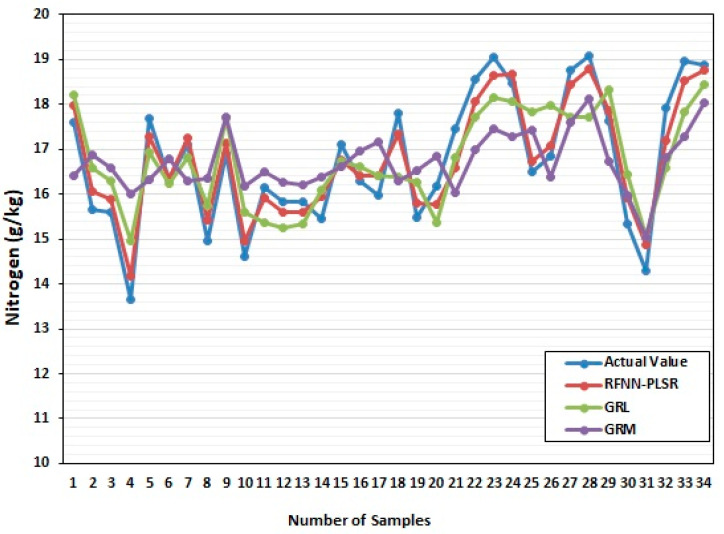
Training model at the maturity stage.

RBFNN model was established and compared GRL and GRM models for leaf area parameters, leaf color parameters, and leaf nitrogen content during the rice-growing stage and the mature stage of RL, as shown in Figure 5 and Figure 6. The GRM model and GRL have a poor fit between the predicted results of nitrogen content and the actual value of nitrogen content, and the RFBNN has a high degree of fit between the predicted value of nitrogen content in RL and the actual value of nitrogen content. In the growing and mature stages, most of RL’s nitrogen content fluctuates between 9~11 g/kg and 15~19 g/kg, respectively. The prediction accuracy of the artificial neural network model is higher than that of the GRL and GRM model in both Figure 3 and Figure 4, and it shows that the RBFNN model is much more reliable, and it fits well with the actual blade, and it is more accurate than GRM model and GRL.

Table 2 shows the comparison of these three models based on the *MAE*, *MAPE*, and *RMSE* (Equation (6)) in which the RFBNN model shows better results during the rice-growing and mature stages are 0.6418%, 0.5399%, 0.0652%, and 0.3540%, 0.1566%, 0.0214%, respectively. In addition, at the growing stage, the RBFNN model shows better results compared to both GRL and GRM, in which *MAE* is reduced by 0.2233% and 0.2785%, respectively.

**Figure 7:**
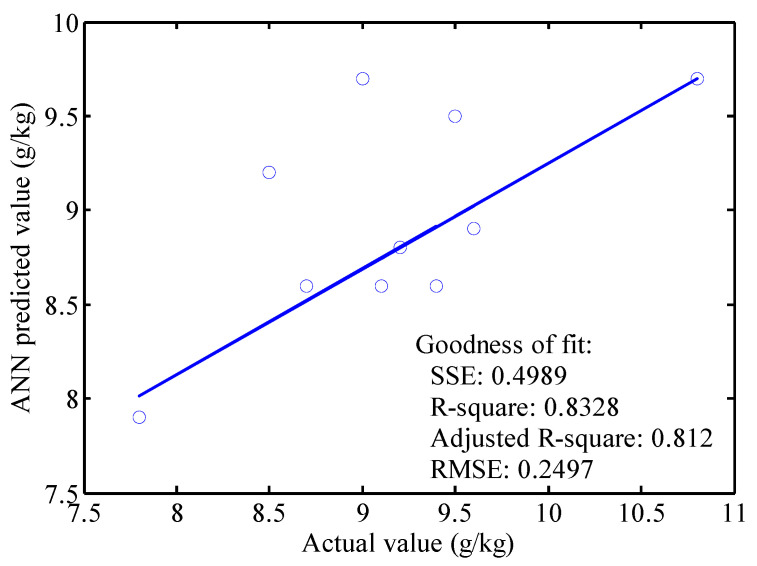
Testing model verification of rice growing stage.

**Figure 8:**
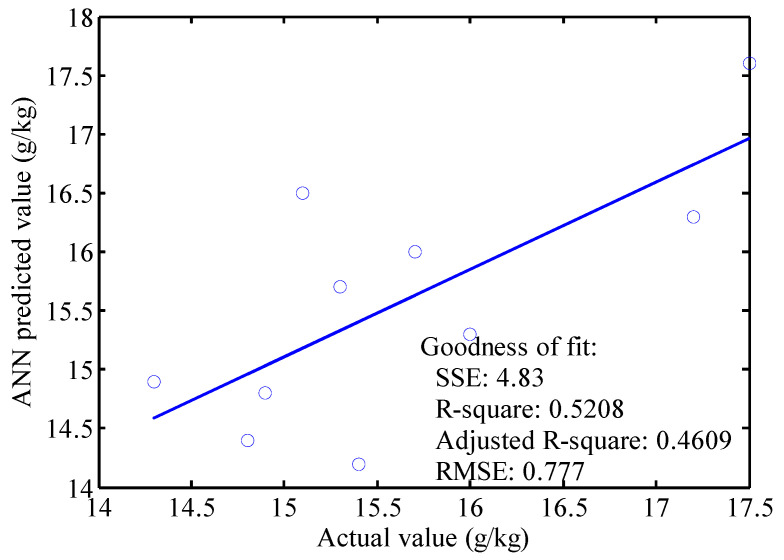
Testing model verification during maturity.

In order to further verify results, 10 samples were used to test the accuracy of the RBFNN model of the leaf color parameters and leaf area of the RL growing stage; moreover, and the leaf nitrogen content of the RL was verified. The verification results showed that the error rate of the predicted nitrogen content of the RL and the measured nitrogen content was between −9.950% and 9.322% during the growing stage. The correlation coefficients for the actual and predicted value of the RL growing and mature stages are 0.6442 and 0.7217. The linear regression of the actual and predicted value of the RL growing and mature stages are shown in Figure 7 and Figure 8, respectively. The *R-square* and *RMSE* for the linear fitting of the actual and predicted value in the rice growing stage are 0.8328 and 0.2497, which means the linear fitting is good. However, the slope of the fitting line is 0.5635, which means the accuracy of this method can be improved in future work. The *R-square* and *RMSE* for the linear fitting of the actual and predicted value in the mature stage are 0.5208 and 0.777, which is worse than that of the rice growing stage. The slope of the fitting line for the mature stage is 0.749. The model’s predicted value is consistent with the test result, which verifies the reliability of the artificial neural network model.

## 4. Conclusions

This paper presented a model called RBFNN-PLSR to predict the Nitrogen content of rice leaves using multispectral images. The RBFNN-PLSR model forecasted that, given the same amount of data, the average relative error percentage of the total nitrogen content in the leaf would be lower, and the prediction accuracy would be greater. Further investigation is essential because the PLSR algorithm was used for optimization. The proposed model can reasonably estimate the nitrogen content in the leaf and provide a scientific basis for developing efficient fertilization management and information-based precision agriculture. The limitation related to preprocessing transformations which affects the model’s prediction performance. Future work will investigate the remaining fertilization components, such as phosphorus in rice, which can affect health. Finally, this model may also be used to continuously monitor the nitrogen nutrition status of rice, which will provide the best basis for performing precise fertilization control.

## Figures and Tables

**Table 1:** Rice varieties.

No.	Accession No.	Origin	Local Name
1	Acc3369	Sarawak	Mansau
2	Acc6891	Sarawak	Biris
3	Acc6893	Sarawak	Padi Wangi
4	Acc7155	Sarawak	Chelom I
5	Acc7156	Sarawak	Chendana
6	Acc5080	Peninsular Malaysia	Chempa (Padi Huma)
7	Acc5101	Peninsular Malaysia	Siong Pelandok
8	Acc5103	Peninsular Malaysia	Anak Cina (H)
9	Acc5105	Peninsular Malaysia	Bongkok
10	Acc6009	Peninsular Malaysia	Mayang Lega
11	Acc9936	Sabah	Janda Muda
12	Acc9953	Sabah	Padi Purak
13	Acc9954	Sabah	Padi Mansud
14	Acc9956	Sabah	Padi Beruang
15	Acc9958	Sabah	Padi Tiga Bulan

**Table 2:** Comparison of the performance of prediction models.

Model Type	Growing Stage			Mature Stage		
	*MAE* (%)	*MAPE* (%)	*RMSE* (%)	*MAE* (%)	*MAPE* (%)	*RMSE* (%)
RBFNN	0.6418	0.5399	0.0652	0.3540	0.1566	0.0214
GRL	0.8651	1.0545	0.0881	0.7944	0.7399	0.0474
GRM	0.9203	1.2395	0.0953	1.0141	1.2272	0.0607

## Data Availability

Data were collected from the Laboratory of Climate-Smart Food Crop Production, Institute of Tropical Agriculture and Food Security, Universiti Putra Malaysia (UPM), 43400 Serdang, Selangor, Malaysia.
